# Smad2 and Smad6 as predictors of overall survival in oral squamous cell carcinoma patients

**DOI:** 10.1186/1476-4598-9-106

**Published:** 2010-05-12

**Authors:** Flavia RR Mangone, Fernando Walder, Simone Maistro, Fátima S Pasini, Carlos N Lehn, Marcos B Carvalho, M Mitzi Brentani, Igor Snitcovsky, Miriam HH Federico

**Affiliations:** 1 Disciplina de Oncologia, Departamento de Radiologia, LIM 24, Hospital das Clínicas da Faculdade de Medicina da Universidade de São Paulo, Avenida Dr Arnaldo 455, São Paulo, Brasil; 2 Departamento de Otorrinolaringologia, Universidade Federal de São Paulo, São Paulo, Brasil; 3 Serviço de Cirurgia de Cabeça e Pescoço, Hospital Heliópolis, São Paulo, Brasil

## Abstract

**Background:**

To test if the expression of Smad1-8 mRNAs were predictive of survival in patients with oral squamous cell carcinoma (SCC).

**Patients and Methods:**

We analyzed, prospectively, the expression of Smad1-8, by means of Ribonuclease Protection Assay in 48 primary, operable, oral SCC. In addition, 21 larynx, 10 oropharynx and 4 hypopharynx SCC and 65 matched adjacent mucosa, available for study, were also included. For survival analysis, patients were categorized as positive or negative for each Smad, according to median mRNA expression. We also performed real-time quantitative PCR (QRTPCR) to asses the pattern of TGFβ1, TGFβ2, TGFβ3 in oral SCC.

**Results:**

Our results showed that Smad2 and Smad6 mRNA expression were both associated with survival in Oral SCC patients. Cox Multivariate analysis revealed that Smad6 positivity and Smad2 negativity were both predictive of good prognosis for oral SCC patients, independent of lymph nodal status (*P *= 0.003 and *P *= 0.029, respectively). In addition, simultaneously Smad2^- ^and Smad6^+ ^oral SCC group of patients did not reach median overall survival (mOS) whereas the mOS of Smad2^+^/Smad6^- ^subgroup was 11.6 months (*P *= 0.004, univariate analysis). Regarding to TGFβ isoforms, we found that Smad2 mRNA and TGFβ1 mRNA were inversely correlated (p = 0.05, R = -0.33), and that seven of the eight TGFβ1^+ ^patients were Smad2^-^. In larynx SCC, Smad7^- ^patients did not reach mOS whereas mOS of Smad7^+ ^patients were only 7.0 months (*P *= 0.04). No other correlations were found among Smad expression, clinico-pathological characteristics and survival in oral, larynx, hypopharynx, oropharynx or the entire head and neck SCC population.

**Conclusion:**

Smad6 together with Smad2 may be prognostic factors, independent of nodal status in oral SCC after curative resection. The underlying mechanism which involves aberrant TGFβ signaling should be better clarified in the future.

## Background

The Smad family of proteins, Smads 1 to 8, are key molecules in Transforming Growth Factor-β (TGFβ) signaling, eventually modulating both TGFβ tumor suppressive and oncogenic effects [1]. Among them, Smad2 and Smad3 are known as receptor regulated Smads (R-Smads) and are phosphorylated in response to TGFβ itself. The phosphorylated protein, in conjunction with the common Smad (Co-Smad), Smad4, translocates to the nucleus eliciting the transcription of other genes [2-4]. The Inhibitory Smads (I-Smads) Smad6 and 7, on the other hand, prevent the activation of R-Smad by phosphorylation and/or interfering with its nuclear translocation [5-7].

Smad signaling seems to be relevant to the pathogenesis of several epithelial cancers. Smad4 and Smad2 functions are disrupted in pancreatic, esophageal, gastric, colon and lung cancer [8-12]. Over-expression of inhibitory Smad6 and Smad7 was described in pancreatic cancer and in pancreatic cancer cell lines [13,14]. Smad2 and 3 present different targets and have distinctive roles, as shown in skin tumors of transgenic mice [15].

Concerning head and neck squamous cell carcinoma (HNSCC), however, data on Smads are still scarce. Studies done with HNSCC samples have shown alterations of individual Smad expression as measured by immunohistochemistry [16,17]. In addition, evidence obtained in *in vitro *studies indicates that Smad signaling may enhance invasiveness in HNSCC [18].

We have previously suggested that, in oral SCC but not in other HNSCC sites, the tumor supressive effect of TGFβ was absent in lymph node positive (pN+) but still present in lymph node negative (pN0) patients [19]. Therefore, we assumed that the extent of expression of individual Smad mRNAs might reflect the degree of TGFβ resistance, and in this way, correlate with progression in oral SCC and consequently with survival. In this work, we found that Smad family mRNA expression was globally increased in HNSCC as compared to adjacent tissue. In addition, among all Smads, Smad2 and Smad6 were suggested to be prognostic markers, correlating with overall survival.

## Patients and Methods

### Patients

Surgical specimens of primary oral SCC were prospectively and sequentially obtained from 48 patients (median age 55 years, range 30 - 86; 43 male and 5 female) with previously untreated, operable HNSCC admitted at the Department of Head and Neck Surgery, Hospital Heliópolis - São Paulo - SP - Brazil. Matched adjacent mucosa, from the resection margin, was obtained from 40 patients. The Smad 1-8 mRNA expression of these and other 35 samples from different head and neck sites was evaluated. The general characteristics of patients are presented in Table 1.

**Table 1:** Clinical Pathological characteristics of studied population.

	Oral Cavity	Larynx	Oropharynx	Hypopharynx	total
**Lymph node status**					
**pN0**	23	11	2	2	**38**
**pN+**	25	10	8	2	**45**
					
**Tumor size**					
**pT1/T2**	17	4	1	0	**22**
**pT3/T4**	31	17	9	4	**61**
					
**Clinical Staging**					
**I/II**	13	4	0	0	**17**
**III/IV**	35	17	10	4	**66**
					
**total**	**48**	**21**	**10**	**4**	**83**

**Oral cavity**: 25 mouth floor, 5 lower gum, 5 retromolar area, 10 tongue border, 1 hard palate, 1 tongue ventricular surface, 1 mouth anterior floor; **Larynx**: 6 aryepiglottic folds, 10 vocal cords, 3 epiglottis, 2 false cord; and **Oropharynx**: 2 glossotonsilar sulch, 6 tonsil, 1 soft palate, 1 vallecula. All 4 **Hypopharynx **tumors were from pyriform sinus.

All specimens were snap-frozen and stored in liquid nitrogen until analysis. Tumor staging was performed according to the Fifth Edition of the UICC TNM Classification of malignant tumors. Patient follow-up ranged from 14.0 to 53.0 months (median 33.0 months). At the last follow-up, among the 83 patients, 30 had local recurrences, 17 had regional recurrences, 44 patients had died and 6 patients were lost to follow-up.

The protocol was approved by the human review boards at the participating institutions and registered at Brazilian National Research Committee (CONEP). All patients provided voluntary written informed consent before enrolment in compliance with the Declaration of Helsinki and its amendments.

### RNA Extraction and Ribonuclease Protection Assay (RPA)

Frozen tissue samples were pulverized and total RNA was obtained by using TRIzol Reagent (Invitrogen, Life Technologies) following the manufacturer's instructions. Detection and quantification of the Smad family members and of the ribosomal protein L32 were carried out with hSmad multiprobe template set (PharMingen's RiboQuant™ Mult-Probe Ribonuclease Protection Assay System) according to the manufacturer's protocol. Briefly, 10 μg of total RNA were hybridized with α^32^P-UTP (GE Healthcare Biosciences - formerly Amersham-Biosciences, St. Giles, UK) labelled riboprobes (8.0 × 10^5 ^cpm per sample) for 16 hours at 56°C, subjected to RNAse A+T1 digestion followed by a phenol-chloroform extraction and ethanol precipitation. The protected double strand RNAs were eletrophoresed in a 5% acrylamide/bis-acrylamide (29:1) urea-containing gel, then the gel was dried and subjected to autoradiography (Hyperfilm, Amersham Biosciences) for 48 hs at -70°C. Specific bands were identified by their distinctive migration pattern as compared to the pattern of undigested probes. Densitometric analysis (ImageMaster VDS software, version 2.0 - Amersham Biosciences) was used for quantification. Each Smad mRNA expression was normalized to L32 housekeeping gene.

### Real-Time Quantitative Reverse Transcriptase PCR (real time QRT PCR)

Five micrograms of total RNA were reverse-transcribed using Random Hexamer primer pre-hit for 10 minutes at 70°C and incubated 10 minutes at room temperature before the addition of the reaction mix (1× Buffer Super Script III, 20 μM of each deoxynucleotide triphosphate, 10 U Super Script III and 0.02 M DTT - Invitrogen, CA, USA). The reaction was performed at 55°C per 50 minutes and interrupted by 15 minutes incubation at 70°C.

Real-time QRT PCR was carried out with SYBR Green dye in a Rotor Gene - RG300 (Corbett Research, DE). Oligonucleotide primers were designed for human TGFβ isoforms and β-actin house keeping using the Primer3 program (Whitehead Institute for Biomedical Research, http://www.bioinformatics.nl/cgi-bin/primer3/primer3_www.cgi), based on its mRNA sequences. The synthesized forward and reverse primer sequences were (IDT, Integrated DNA Technologies, IA, USA): β-actin (NM_001101.3: fw 5'AGAAAATCTGGCACCACACC3' and rev 5'AGAGGCGTACAGGGATAGCA3'); TGFβ1 (NM_000660.4: fw 5'CCCTGGACACCAACTATTGC3' and rev 5'TGCGGAAGTCAATGTACAGC3'; TGFβ2 (NM_003238.2: fw 5'GAGTGCCTGAACAACGGATT3' and rev 5'TTCACAACTTTGCTGTCGATG3'); TGFβ3 (NM_003239.2: fw 5'TGATCCAGGGGCTGGCGGAG3' and rev 5'GGGTTGGGCACCCGCAAGA3').

The PCR reaction mixture, performed with 100 ng of cDNA, was: 1.5× SYBR Green I nucleic acid gel stain (Molecular Probes, OR, USA), 1× PCR Buffer, 2 mM Magnesium Chloride, 0.2 mM of each deoxynucleotide triphosphate, 1.5 U of Platinum Taq DNA Polymerase, 0.5 mg/mL Bovine Serum Albumin Acetylated (Promega, WI, USA), 5% of DMSO (Sigma, CA, USA), 0.2 μM of each primer in a total volume of 20 mL in Molecular Biology Grade Water (Invitrogen, Life Technologies, CA, USA). These experiments were performed in duplicate. The thermal cycling included an initial denaturation step of 5 minutes at 95°C followed by 35 cycles of 15 seconds at 95°C, 1 minute at 60°C and 1 minute at 72°C. Melting analysis was performed by heating the reaction mixture from 74 to 99°C at a rate of 0.2°C/second. Threshold cycle (Ct) and melting curves were acquired by using the *"quantitation" and "melting curve" *program of the Rotor gene 6 software version 6.0 Corbett Research (Corbett Research, DE). Only genes with clear and single melting peaks were taken for further data analysis. Samples with irregular melting peaks were excluded from the calculation. The threshold was set manually, using identical threshold levels for one gene in all analyzed samples. Reaction efficiency was established for each set of primers, after quantification of four different dilutions of a reference cDNA.

The Ct value of three targets genes (TGFβ1, TGFβ2 and TGFβ3) was normalized to the reference gene Ct (β-actin) and the relative quantification was performed according to Pfaff mathematical model [20].

### Statistical analysis

Comparisons between groups were performed by the paired Wilcoxon test, when appropriate. For survival and Spearman's correlation analysis, patients were categorized as positive (Smad^+^, TGFβ^+^) or negative (Smad^-^, TGFβ^-^) according to the median relative expression of each (above or equal/below the correspondent median tumor expression). Overall survival (OS) and disease free survival (DFS) were considered from the day of the surgery to date of death or the date in which recurrence was detected, by means of physical examination or imaging. Survival curves were estimated using the Kaplan Meier method and compared using the univariate Log Rank test. A Cox multivariate analysis was performed to identify independent predictors of survival. All statistics were done using SPSS 10.0 statistical software (SPSS Inc., Chicago, IL). Differences were considered statistically significant for *P *value ≤ 0.05.

## Results

### Smad mRNA expression in oral SCC

**Figure 1:**
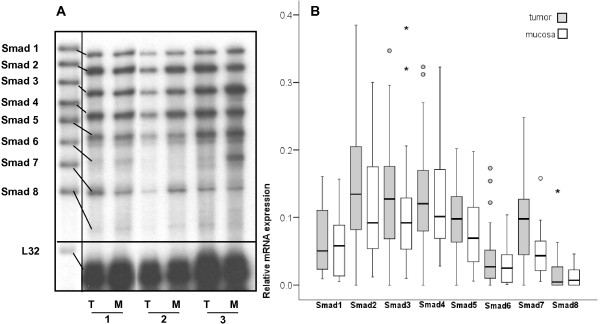
**Smad mRNA expression in oral SCC: A**. RPA representative assay. Lane 1: riboprobe; represented as pairs: T-tumor M- matched adjacent mucosa; L32: housekeeping gene. **B**. Boxplot representing Smad1-8 mRNA expression. *Boxes: *25^th^, 50^th ^and 75^th ^percentiles; *bars*: 10^th ^and 90^th ^percentiles; **°**: outlier values (1.5-3 box-lengths from 75^th ^percentiles); _*_: extreme values (>3 box-lengths from 75^th ^percentiles).

In this study, we determined Smads (Smad1 to 8) mRNA expression in 48 primary tumors (Table 1) from patients with oral SCC submitted to curative ressection (see representative assay in figure 1A). Quantification of the RPA signals, normalized to L32 mRNA, revealed that, up to 91% of the tumors expressed all Smad mRNAs, except for Smad8 mRNA, detected in 73% of the tumors. In parallel, 100% of the 40 available specimens of adjacent mucosa expressed Smads 1 to 7, and Smad8 mRNA expression was detected in 52.5%. The distribution and median of Smads expression are shown in Figure 1B.

Paired analysis revealed that oral SCC express more Smad2, 3, 5 and 7 mRNAs than matched mucosas (*P *< 0.05, Table 2). When clinical-pathological features such as lymph node status, tumor size, differentiation degree, pathological staging, age, gender and smoking degree were considered, no statistically significant differences were found.

**Table 2:** Smad mRNA expression in head and neck SCC and adjacent mucosa.

		All patients n = 65	Oral Cavity n = 40	Larynx n = 15	Oropharynx n = 8
**Smad1**	**T**	0.06 ± 0.06	0.06 ± 0.05	0.07 ± 0.06	0.06 ± 0.10
	**M**	0.05 ± 0.05	0.06 ± 0.05	0.05 ± 0.04	0.03 ± 0.05
		*P *= 0.017*	*P *= 0.209	*P *= 0.256	*P *= 0.012
					
**Smad2**	**T**	0.15 ± 0.10	0.16 ± 0.10	0.17 ± 0.10	0.13 ± 0.10
	**M**	0.11 ± 0.09	0.11 ± 0.08	0.15 ± 0.10	0.08 ± 0.11
		*P *= 0.002*	*P *= 0.020*	*P *= 0.532	*P *= 0.036*
					
**Smad3**	**T**	0.15 ± 0.11	0.13 ± 0.08	0.18 ± 0.13	0.18 ± 0.16
	**M**	0.11 ± 0.09	0.10 ± 0.08	0.15 ± 0.12	0.06 ± 0.08
		*P *= 0.001*	*P *= 0.028*	*P *= 0.047*	*P *= 0.017*
					
**Smad4**	**T**	0.13 ± 0.09	0.13 ± 0.08	0.15 ± 0.09	0.12 ± 0.12
	**M**	0.11 ± 0.08	0.12 ± 0.07	0.13 ± 0.08	0.09 ± 0.11
		*P *= 0.051*	*P *= 0.202	*P *= 0.733	*P *= 0.017*
					
**Smad5**	**T**	0.10 ± 0.06	0.10 ± 0.05	0.12 ± 0.08	0.08 ± 0.07
	**M**	0.08 ± 0.06	0.08 ± 0.05	0.01 ± 0.07	0.04 ± 0.06
		*P *= 0.001*	*P *= 0.006*	*P *= 0.798	*P *= 0.036*
					
**Smad6**	**T**	0.03 ± 0.04	0.04 ± 0.04	0.03 ± 0.03	0.01 ± 0.01
	**M**	0.02 ± 0.02	0.03 ± 0.03	0.10 ± 0.01	0.01 ± 0.02
		*P *= 0.072*	*P *= 0.132	*P *= 0.334	*P *= 0.833
					
**Smad7**	**T**	0.08 ± 0.06	0.09 ± 0.06	0.08 ± 0.06	0.04 ± 0.03
	**M**	0.04 ± 0.03	0.05 ± 0.03	0.03 ± 0.04	0.03 ± 0.03
		*P*<0.0001*	*P*<0.0001*	*P *= 0.002*	*P *= 0.123
					
**Smad8**	**T**	0.02 ± 0.03	0.02 ± 0.03	0.02 ± 0.03	0.01 ± 0.01
	**M**	0.01 ± 0.02	0.01 ± 0.01	0.02 ± 0.03	0.01 ± 0.01
		*P *= 0.378	*P *= 0.389	*P *= 0.875	*P *= 0.496

**T**: tumor, **M**: adjacent mucosa. Values are presented as median ± standard deviation, * significant differences by Wilcoxon test.

**Figure 2:**
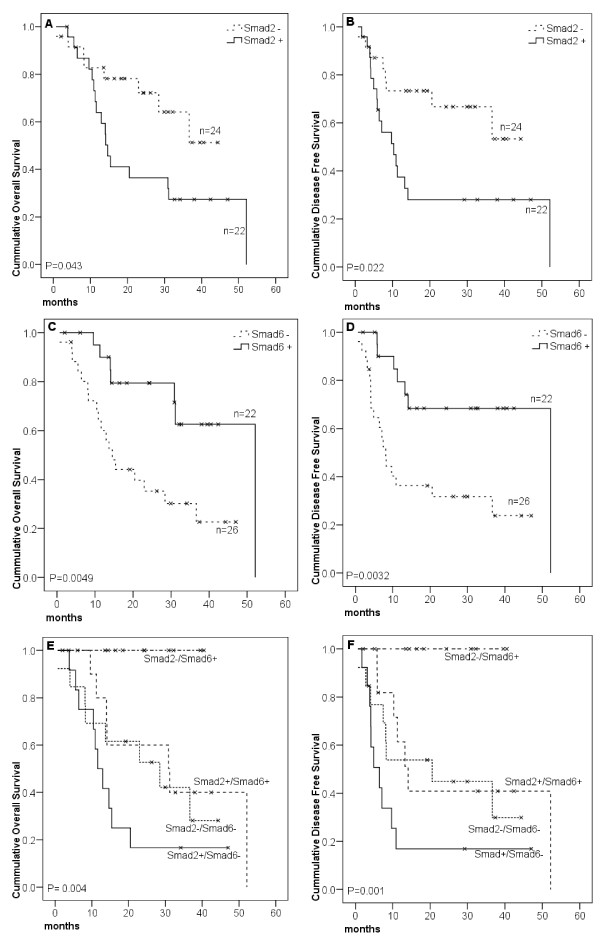
**Kaplan-Meier survival curves of oral cavity SCC patients grouped according to Smad expression**. Patients were categorized as positive (above) or negative (equal or below) according to median Smad expression in tumors. Log Rank test was performed for curves comparison. In **E **and **F**, patients were grouped according to the co-expression of Smad2 and Smad6. Smad2^+^/Smad6^-^: n = 13; Smad2^+^/Smad6^+^: n = 11; Smad2^-^/Smad6^-^: n = 13; Smad2^-^/Smad6^+^: n = 11.

In survival analysis, Smad2^- ^patients presented longer median OS as compared to Smad2^+ ^patients (median OS not reached and 14.6 months, respectively), while the mOS for the entire oral SCC group was 31 months, a similar behaviour being found for mDFS (Figure 2A and 2B). Similarly, Smad6^+ ^patients presented a median OS three times longer than Smad6^- ^patients (52 and 14.6 months, respectively). Also, the Smad6^+ ^mDFS was 52 months, six times longer than that of Smad6^- ^patients (Figure 2C and 2D). According to lymph node status, the mOS was 37 months in pN0 patients and 14 months in pN+ patients, with survival rates of 61% in pN0 patients (n = 23) and 36 in pN+ patients (n = 25, p < 0.05).

By Cox regression multivariate analysis, those two markers, Smad2 and Smad6, were shown to be prognostic factors in oral SCC, independent of lymph nodal status (Table 3). In line with this, even with a small population, pN+ patients who were Smad6^+ ^presented 31 months mOS, three times longer than that presented by Smad6^- ^patients (11 months). Accordingly, in the pN0 subgroup, Smad6^- ^patients had 23 month mOS, shorter than that of Smad6^+ ^patients who did not reach mOS (*P *= 0.009). The same seems to occur with simultaneously Smad2^+ ^and pN+ patients who presented a poorer prognosis as compared to Smad2^-^/pN+ patients who did not reach mOS. Consonant with this, patients who were Smad2^-^/Smad6^+ ^simultaneously did not reach mOS and mDFS whereas Smad2^+^/Smad6^- ^patients presented mOS 12 months and mDFS 6 months (*P *= 0.0044, *P *= 0.0012, respectively, Figure 2E, F).

**Table 3:** Cox multivariate analyses of survival prediction in oral SCC.

Variable	Hazard ratio (95% CI)	*P *value
Smad2	0.374 (0.155 - 0.906)	0.029
Smad6	4.153 (1.613 - 10.692)	0.003
pN	0.598 (0.233 - 1.531)	0.284
pT	0.557 (0.197 - 1.575)	0.270

CI - confidence interval

### Smad mRNA expression in larynx SCC and in other HNSCC subsites

Except for Smad6 (90%) and Smad8 (86%), Smads were expressed in 100% of larynx SCC. In matched adjacent mucosa, Smad6 was expressed in 73%, Smad7 in 93% and Smad8 in 67%, the others being expressed in 100% of samples.

**Figure 3:**
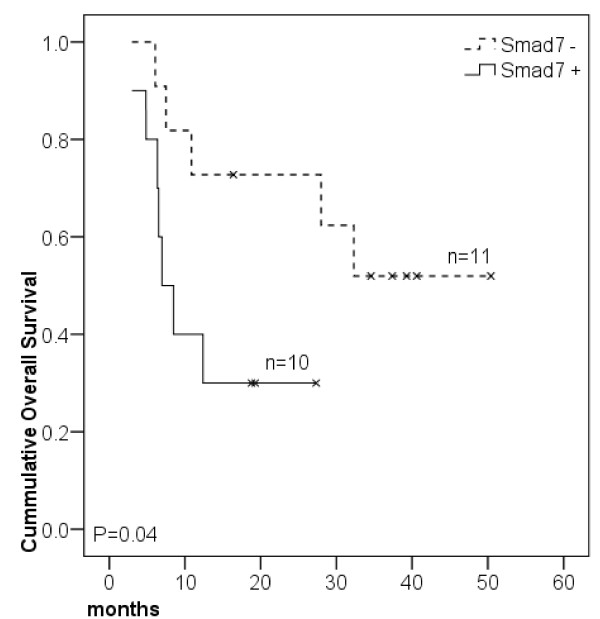
**Kaplan-Meier survival curves of larynx SCC patients grouped according to Smad expression**. Patients were categorized as positive (above) or negative (equal or bellow) according to median tumor expression. Log Rank test was performed for curves comparison.

Paired analysis revealed that only Smad7 was overexpressed in tumors (0.08 ± 0.06) as compared to adjacent mucosa (0.03 ± 0.04, *P *= 0.002). This marker was the only one to correlate with survival in this subset of patients, with a survival advantage observed in Smad7^- ^patients (mOS not reached) over those who were Smad7^+ ^(mOS 6.97 months, *P *= 0.04, Figure 3). The survival rate of Smad7^- ^and Smad7^+ ^patients was 54.5% and 30% respectively. When clinical-pathological features as lymph node status, tumor size, differentiation degree, pathological staging, age, gender and smoking degree were considered, no statistical difference was found related to Smad7 status.

In oropharynx SCC, even with a small sample size, paired analysis showed Smad overexpression for Smad2 to 5, while in the entire HNSCC population, a significant difference was not achieved only for Smad8, confirming that differences between HNSCC subsites must be considered (Table 2).

Concerning Smad expression, OS and DFS, no correlations were found as revealed by Log Rank analysis, in the HNSCC population as a whole.

### TGFβ isoforms mRNA expression profile in Oral SCC

We assessed the three TGFβ isoforms using real-time QRT PCR in 35 available oral SCC. TGFβ1 mRNA expression was observed in 100% of samples (mean ± standard deviation: 0.82 ± 0.68, median: 0.57), while TGFβ2 (94%) and TGFβ3 (97%) isoforms were detected in almost all tumors (TGFβ2:1.24 ± 1.82, 0.72; TGFβ3: 2.12 ± 3.58, 1.15).

No statistically significant associations were found between TGFβ isoforms and clinical-pathological characteristics such as pT, pN, pathological staging and histological differentiation. After categorization according to median mRNA expression, the observed positivity of each isoform was 23%, 37% 49% for TGFβ1, TGFβ2 and TGFβ3, respectively.

The correlations among categorized Smad2, Smad6, TGFβ1, TGFβ2 and TGFβ3 were tested by Spearman's rho test. Results revealed that Smad2 was inversely correlated to TGFβ1 (*P *= 0.05, R = -0.33). Among the eight TGFβ1^+ ^tumors, seven (87.5%) were also Smad2^-^, while in TGFβ1^- ^subgroup an equal distribution between Smad2^- ^(48%) and Smad2^+ ^(52%) was observed.

## Discussion

In this study, we provide evidence that Smads may have a key role in head and neck cancer, influencing survival. Specifically, we show, prospectively, that high Smad6 mRNA expression and low Smad2 mRNA correlate with better survival in oral SCC patients submitted to curative surgery, independent of nodal status. In addition, Smad7 high mRNA expression correlated with shorter survival in patients with larynx SCC submitted to curative surgery. The fact that these correlations were restricted to specific tumor locations supports the hypothesis that underlying biological heterogeneity exists between different subsites within head and neck.

TGFβ is known as a potent tumor supressor in normal epithelial cells and in early-stage tumors but during tumor progression it becomes an oncogenic factor, mainly in advanced tumors [21]. Since Smad6 has a blocking effect on TGFβ signaling either by direct binding to the TGFβ receptor, by competing with Co-Smad for R-Smad complex formation or by targeting receptor for degradation [6,7,22,23], we can speculate that this effect on survival occurs because Smad6 would inhibit the TGFβ tumorigenic signaling, thus favouring a better outcome. The favorable prognosis presented by Smad6^+ ^oral SCC patients agrees with that described in 115 esophageal SCC using immunohistochemistry [24]. Reinforcing this idea, we found that even in the poor prognosis group, pN+, the presence of Smad6^+ ^was associated with a survival advantage similar to that of pN0 patients.

Smad2 is the classically R-Smad of the TGFβ pathway [1], our own data suggest Smad2 negativity reinforces the effect of Smad6 on survival of oral SCC. Smad2 may be a key factor for the interruption of TGFβ tumorigenic signaling in this group of patients, together with Smad6. In accordance with that, we found TGFβ1 positive tumors were also Smad2 negative. Our finding regarding the association between lack of Smad2 expression and a favourable clinical outcome is in disagreement with previous studies done in head and neck cancer as well as other tumor types. In oral squamous cell carcinoma, a study involving 125 patients suggested a link between decreased expression of both activated Smad2 (p-Smad2) and TGFβ receptor II (TβR-II), with aggressive tumor features, which suggests TGFβ signaling exerts a protective role possibly through Smad 2 [25]. In accordance, the Smad expression profile assessed by tissue array in 170 head and neck squamous cell carcinoma pointed to the loss of TGFβ/Smad2 signaling as a possible cause of adverse outcome [17]. A protective role of Smad 2 was also suggested for esophageal, colon and breast cancer [26-28]. In line with this effect, others have identified a missense mutation of Smad2 in the squamous cell line SCC-15 suggesting that the loss of Smad 2 may be part of head and neck carcinogenesis [29].

There are, however, several studies in line with our data. Matrix metalloproteinases play roles in cancer progression by degrading the extracellular matrix and basement membrane. TGFβ1 signaling induces MMP-9 expression via Smad 2/3 [30]. Accordingly, the TGFβ1/Smad 2/3 axis regulates MMP-9 expression through the transcriptional factors Snail and Ets-1, contributing to oral cancer progression [31]. In addition, the metastasis-associated protein metastatin can physically and functionally interact with Smad2/3, and enhance TGFβ mediated MMP-9 induction [32]. Activation of Smad 2/3 signaling has also been linked to enhancement of MMP-13 expression and invasion of head and neck squamous carcinoma cells [18].

Smad 2 accumulation seems to parallel an elevation of H-ras, both of which are essential for epithelial mesenchymal transition (EMT), an essential step during carcinogenesis. Having undergone EMT, others have shown that fibroblastoid carcinoma cells with elevated levels of activated Smad2 gain the capability to spread to a wide variety of tissues by a further increase in Smad2 expression [33]. In addition, a prominent expression of alpha(v)beta(6) integrin at tumor stroma interface in xenograft model, which resembles human head and neck carcinomas, has been connected with cancer progression. Alpha(v)beta(6) interacts with TGFβ, as an Alpha(v)beta(6) blocking antibody can inhibit TGFβ mediated Smad2/3 phosphorylation, which leads to inhibition of tumor growth *in vivo*, suggesting a role for the microenvironment for this effect [34].

**Figure 4:**
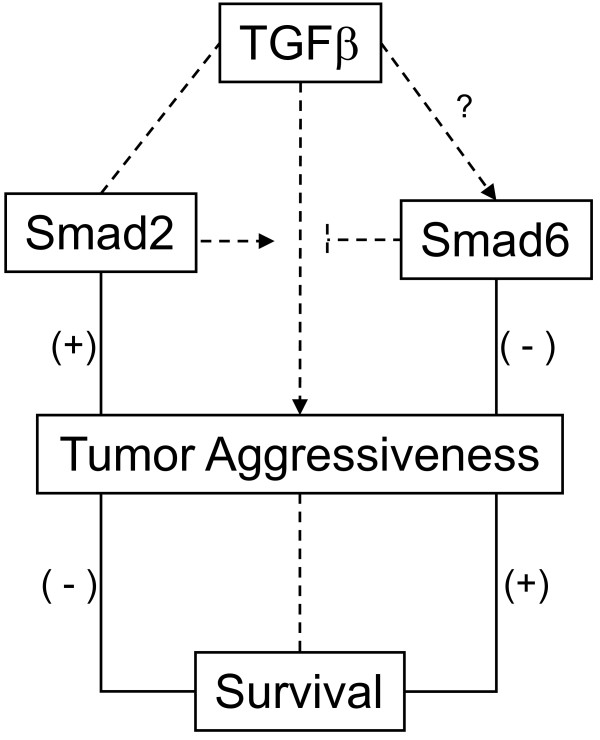
**Hypothesis scheme of Smad influence on survival**. TGFβ1 upregulation is associated with tumor agressiveness and poor patient survival. Smad2 and Smad6 have opposite roles in this process. Full and dashed lines represent our own findings and previous published data, respectively.

It is not easy to reconcile these conflicting results regarding the role of Smad2, in head and neck tumors, since they reflect the dual roles of TGFβ itself, acting both as a tumor suppressor and a tumor enhancer, depending on the context. What is not clear, however, is what constitutes this context [35]. In our head and neck patient series, Smad2 seems to be a TGFβ signaling node that enhances tumor aggressiveness (Figure 4). An intriguing possibility, that merits further study, is that HPV infection may be interacting with TGFβ network as shown in cervical carcinoma [36].

Taking into account that Smad6 is at the crossroad of many signaling pathways, being regulated not only by family members of bone morphogenetic protein (BMP) and TGFβ, but also by epidermal growth factor and Ras/MAPK [6,7,37], its expression may be key to shift signaling from oncogenesis to tumor supression, towards or against proliferation, independent of Smad3 and Smad4, at least in oral SCC (Figure 4).

If we consider Smad6 and Smad7 as interchangeable TGFβ blocking Smads, our data in oral SCC is also in keeping with others, showing that a combination of increased immunohistochemical expression of Smad7 and decreased Smad4 expression are markers of good prognosis in gastric cancer, with 67.5% survival rate versus 52.2%, (*P *= 0.0011) [38]. However, data concerning Smad7 seems to be more controversial since our own present data indicates that Smad7 mRNA low expression correlates with shorter survival in larynx SCC (Figure 3). In agreement with our results, patients with colon and gastric cancers, with Smad7 gene deletion or low Smad7 protein expression, were described as having prolonged survival as compared to patients with higher Smad7 expression [39,38].

Concerning the lack of influence of Smad4 mRNA expression on patient survival in our study, data in the literature suggests that there is no single rule for this. Low Smad4 expression, detected by immunohistochemistry, and poorer five-year survival was shown in 249 patients with advanced gastric cancer and in 258 esophageal squamous cell carcinomas (*P <*0.05) [9,10]. In colon cancer, such an influence was not shown, which is in accordance with our present data [39]. Taking into account that Smad4 mRNA was the most ubiquitous among Smads we can argue that perhaps the amount of Smad4 was not a crucial element for these patients.

Finally, we found higher Smad mRNA expression in SCC primary tumors as compared to adjacent tissue in agreement with previously published data, showing that HNSCC cell lines present multiple defects in TGF-beta signaling [40]. In HNSCC samples, data obtained from 170 tumors using tissue array technology, shows expression of Smad2, Smad3 and Smad4 proteins [17]. Another small study comparing the expression between tumor and adjacent tissue did not find differences at least with respect to Smad4, Smad6 and Smad7 protein expression in 13 head and neck SCC tumors [16].

To sum up, our results suggest that increased Smad6 mRNA expression and low Smad2 mRNA expression might be markers of better outcome in oral SCC but not in larynx cancer submitted to curative surgery. These effects may be linked to aberrant TGFβ signalling. In larynx cancer, a similar relationship was found for Smad7 mRNA low expression. Our findings need to be validated in larger prospective studies, and may in the future, help to stratify candidate patients for adjuvant treatment in head and neck cancer.

## Conclusions

TGFβ is classically known as tumor supressor in normal epithelial cells that turns into a malignant factor during tumor progression favoring tumor growth and metastasis. Here we propose that the interruption in TGFβ tumorigenicity by Smad2 dowregulation or by Smad6 overexpression confers a better outcome. Although both might be considered as prognostic markers, the molecular mechanism envolved in this process is not clear. Further studies are warranted to explore the mechanisms involved.

## Competing interests

The authors declare that they have no competing interests.

## Authors' contributions

FRRM carried out RNAse protection assay and Real-Time QRTPCR, was involved in the study design, data management, statistical analysis and manuscript preparation, FSP responsible for primer designing, real-time QRTPCR support, helped in statistical analysis and revised the manuscript SM helped in statistical analysis and revised the manuscript; SI oversaw the statistical analysis and helped draft the manuscript; CL, FW and MBC were the head and neck surgeons in the team, who collected samples, all clinical data and were involved with data management; MMB helped to draft the manuscript; MHHF conceived and was responsible for coordination of the study, and was responsible for the study funding through FAPESP. All authors read and approved the final manuscript.
